# Online availability of fish antibiotics and documented intent for self-medication

**DOI:** 10.1371/journal.pone.0238538

**Published:** 2020-09-03

**Authors:** Weiwei Zhang, Austin Williams, Nicole Griffith, Jessica Gaskins, P. Brandon Bookstaver

**Affiliations:** 1 Department of Clinical Pharmacy, Beijing Tsinghua Changgung Hospital, School of Clinical Medicine, Tsinghua University, Beijing, China; 2 University of South Carolina College of Pharmacy, Columbia, South Carolina, United States of America; 3 University of Virginia Health, Charlottesville, Virginia, United States of America; 4 South Carolina Aquarium, Charleston, South Carolina, United States of America; 5 Prisma Health Richland, Columbia, South Carolina, United States of America; Nitte University, INDIA

## Abstract

Self-medication and antibiotic utilization without healthcare oversight may lead to delayed appropriate treatment, transmission of communicable infections, untoward adverse events, and contribute to antimicrobial resistance. Previous data suggest people obtain over-the-counter (OTC) animal antibiotics for their personal use. This study examined the availability of OTC fish antibiotics online and the documented intent for self-medication. The authors conducted a web-based cross-sectional study using Google search engine to identify vendor websites selling fish antibiotics in the United States. Vendor websites were included if product information, consumer reviews, and comments were publicly available. Nine fish antibiotics were chosen due to their possibility of having consequences to human misuse. The cost and availability of fish antibiotics was recorded. The proportion of reviews and comments related to human consumption was calculated. Consumer review traffic based on “likes” and “dislikes” received was compared between human- and non-human consumption-related reviews. Selected fish antibiotics were purchased and evaluated for physical appearance and compared to FDA-approved available equivalents. We found 24 website vendors with online ordering available for OTC fish antibiotics. Cost varied significantly by antibiotic and quantity ranging from USD $8.99 to $119.99. There were 2,288 reviews documented for the 9 selected antibiotics being sold. Among consumer reviews, 2.4% were potentially associated with human consumption. Human consumption-related reviews constituted 30.2% of all “likes” received and 37.5% of all “dislikes” received. Human consumption-related reviews received an average of 9.2 likes compared to 0.52 likes for non-human consumption-related reviews. The 8 fish antibiotics purchased were consistent with FDA-approved equivalents in physical appearance. Although infrequent, antibiotics intended for fish use are being purchased online without a prescription for self-medication to circumvent professional medical care. Reviews related to human consumption generate significant online traffic compared to reviews unrelated to human consumption.

## Introduction

Self-medication is defined as the use of over-the-counter (OTC) or prescription medication to treat self-diagnosed disorders, symptoms, or illnesses [1, 2]. Patient self-medication with antibiotics is an inappropriate medication utilization of medication that may produce unwarranted allergic reactions, drug interactions, adverse drug events, and excessive costs [3–5]. Furthermore, this inappropriate antibiotic use can prevent a correct diagnosis, allow for the spread of infection, delay appropriate therapy, and may contribute to antibiotic resistance [3–5]. Self-medication with antibiotics occurs across the globe, and has become a more common phenomenon in the United States (US) [1, 6–12]. A recent review indicated that the prevalence of nonprescription antibiotic use varied from 1% to 66%, depending on population characteristics studied [13].

The availability of OTC antibiotics without a prescription in some countries is a known contributing factor to self-medication. Recent systematic reviews indicate that antibiotics can be purchased without a prescription in many developing countries across the world, despite regulations prohibiting this practice [14, 15]. Online purchases have become an important source of antibiotics without a prescription in the US and United Kingdom (UK) as well [16, 17]. In 2017, 45% of online pharmacies in the UK sold antibiotics without a prescription, 80% of which required consumers to choose the antibiotic agents, dose, and quantity [17]. Another study found that 36.2% of antibiotics sold online in the US were sold without a prescription [16]. In the present COVID-19 era or in an analogous scenario with high rates of unemployment and quarantine restrictions, persons seeking antibiotics online may increase as some avoid in-person visits. Patients consuming antibiotics purchased online without a valid prescription are at additional risk of using the incorrect medication, dose, or duration as well as receiving a poor-quality product lacking official validation of contents.

Recent studies have shown that veterinarian-prescribed antibiotics or antibiotics intended solely for animal use have become a source for self-medication including among members of the US Armed Forces and some underrepresented ethnic communities [18, 19]. Zoorob and colleagues found that 4% of non-prescription antibiotics use were veterinarian-prescribed [19]. Access to these antibiotics intended for animal use appears to be more so driven by OTC availability in street-side businesses (i.e. pet stores) or online marketplaces. In contrast to antibiotics obtained for dogs and cats, the purchase of fish antibiotics in the US does not require pet prescription information, and the products are not FDA-regulated [20]. There are an estimated 13.1 million American households that own a pet fish, creating a significant market for fish medications [21]. In 2018, the FDA released a formal statement, “Ornamental Fish Drugs and You,” to warn the public against utilizing medications intended for animal use. The statement highlights ornamental fish antibiotics have not been approved, conditionally approved, or indexed by the FDA, and the illegality of marketing them for human use [20]. The availability and use of fish antimicrobials was recently highlighted in the global news after a man in the US died from ingesting chloroquine phosphate for fish aquariums in an attempt to prevent COVID-19 [22].

The online availability of OTC fish antibiotics and the intent of consumers purchasing these antibiotics has not been quantified. The purpose of this study was to assess online availability of OTC fish antibiotics and describe their intended use for self-medication through publicly available comments and product purchase reviews.

## Materials and methods

**Fig 1:**
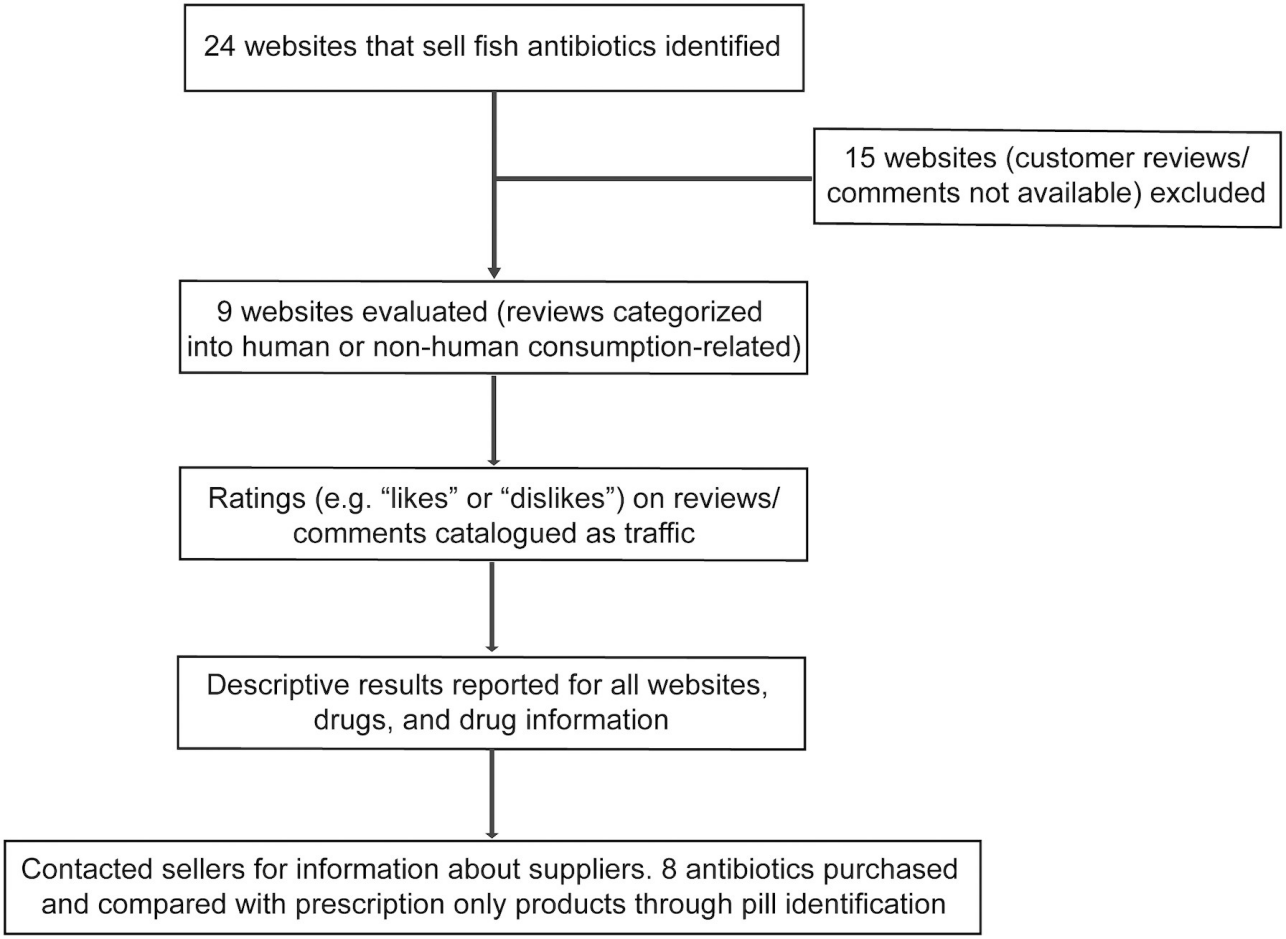
Study methodology.

The authors conducted a prospective, cross-sectional assessment of all website and online vendors selling fish antibiotics between August and September of 2019. The primary objective of this study was to identify the proportion of comments and reviews on online fish antibiotics associated with human consumption. The secondary objectives were to evaluate the traffic and attention received by reviews and comments associated with human consumption versus comments not associated with human consumption. Traffic and attention were measured by the number of reviews, “likes,” and “dislikes” associated with each review/comment. Additionally, the authors sought to evaluate the physical appearance of commonly sought online fish antibiotics and compare them with FDA-approved equivalents. Using the Google^®^ search engine and the key words “fish antibiotics” and “online (English only),” vendors and websites selling fish antibiotics were identified. Vendor websites were included if product information (e.g. pricing, quantity), consumer reviews, and comments were publicly available. Online vendors who do not provide shipment to the US were excluded. Other antimicrobials (e.g. antifungals) were not investigated. Investigators screened the fish antibiotics sold on every website and the top 9 antibiotics that could be commonly used for human consumption were determined. Of note, these antibiotics were officially sold for the intended treatment of ornamental fish and domestic fish tanks. The cost and quantity supplied of fish antibiotics was recorded. The reviews and comments left by consumers who purchased fish antibiotics were read and categorized as either related or unrelated to human consumption (Fig 1). To determine if possibly purchased for human consumption, the following criteria were used to evaluate reviews and comments: (a) Explicitly stated purchased antibiotics were for human consumption. (b) Suggested purchased antibiotics were used for self-medication in a concealed manner (e.g. to treat an infectious disease that only humans could have, purchased due to lack of medical insurance or can’t afford the cost to see a doctor). (c) Suggested that fish antibiotics were the same as human antibiotics and can or should be used for humans. If the verbiage was ambiguous or non-descript (e.g. “The product worked great;” “worked as expected”), the comments were assumed to be non-human related. The proportion of reviews and comments related to human consumption was calculated. All online reviews were conducted by two investigators (PBB, ZW). Discordant reviews were discussed and allocated based on consensus. Eight fish antibiotics (sulfamethoxazole/trimethoprim, cephalexin, amoxicillin, metronidazole, penicillin, ciprofloxacin, clindamycin and doxycycline) were obtained and compared with FDA-approved human equivalents using a commercially available pill identification program (Lexicomp Inc^®^). One fish antibiotic (erythromycin) was not analyzed because fish erythromycins being sold were powder-formed and it was hard to compare them with FDA-approved equivalents using pill identification programs.

### Statistical analysis

Descriptive statistics were used to estimate the prevalence of human related consumption. Data were checked for normality and t-test analyses were conducted to compare traffic between the human consumption-related and non-human consumption-related reviews.

## Results

The authors identified 24 website vendors with online ordering available for OTC fish antibiotics. Nine vendor websites (S1 Table) were included and 15 websites were excluded due to the consumer reviews not existing or not being publicly available. Nine fish antibiotics were selected and analyzed based on their common use in the treatment of human infections and the possibility of having negative repercussions as a result of self-medication (Table 1).

**Table 1:** Baseline information of 9 reviewed antibiotics.

Names of fish antibiotics	Numbers of the reviews	Price range (USD$)			
		Lowest price	Corresponding specifications	Highest price	Corresponding specifications
Amoxicillin	1314	8.99	250mg*30 Tab	75.6	4*500mg*100 Cap
Cephalexin	445	11.99	250mg*30 Cap	40.99	500mg*100 Cap
Metronidazole	229	13.99	500mg*12 Packets	89.99	500mg*100 Cap
Ciprofloxacin	89	27.99	250mg*30 Tab	119.99	500mg*100 Tab
Penicillin	91	13.49	250mg*30 Tab	54.99	500mg*100 Tab
Clindamycin	40	20.99	150mg*30 Cap	54.99	150mg*100 Cap
Doxycycline	36	19.99	100mg*12 Packets	99.99	100mg*100 Cap
Sulfamethoxazole, Trimethoprim	32	18.99	Sulfamethoxazole 800 mg/Trimethoprim 160 mg*30 Tab	42.99	Sulfamethoxazole 800 mg/Trimethoprim 160 mg*100 Tab
Erythromycin	12	19.99	250mg*12 Packets	54.99	250mg*60 Packets

**Fig 2:**
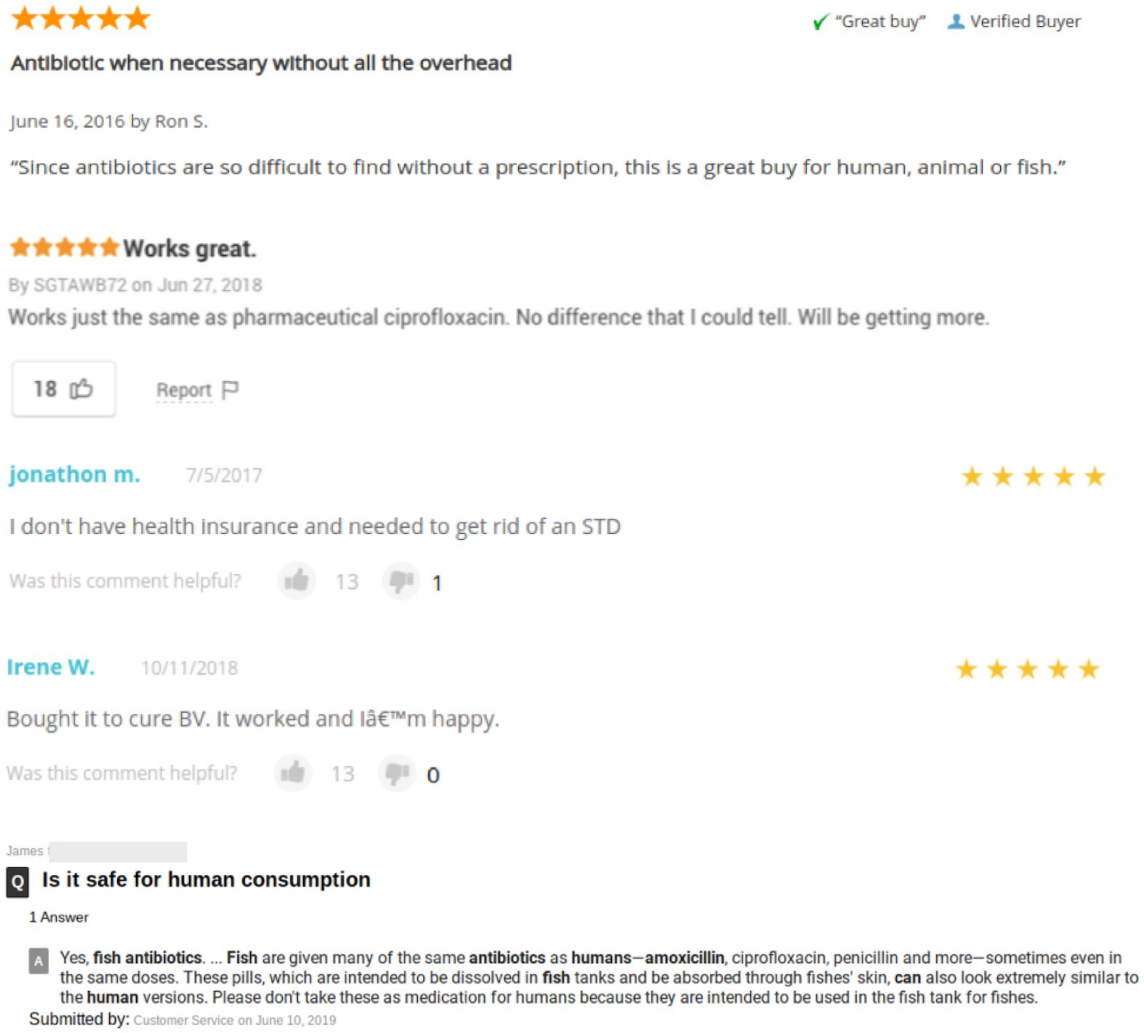
Reviews on self-medication with fish antibiotics.

As shown in Table 1, the cost of fish antibiotics varied significantly between antibiotics and quantities, ranging from $8.99 to $119.99. Consumer review traffic and proportion of reviews related to human consumption are shown in Table 2. There were 2,288 reviews documented for the 9 selected antibiotics being sold. Among consumer reviews, 55 (2.4%) of them were potentially associated with human consumption. There was no statistical difference between the numbers of human consumption-related reviews and non-human consumption-related reviews (p = 0.10). Human-consumption related reviews constituted 30.2% of all “likes” received and 37.5% of all “dislikes” received among all reviews. Human consumption-related reviews received an average of 9.2 likes per review (55 reviews received 506 likes) compared to 0.52 likes per review (2,233 reviews received 1168 likes) for non-human related reviews. Fish antibiotics with a high percentage of human consumption-related reviews are as follows, erythromycin (8.3%), clindamycin (5%), ciprofloxacin (3.4%), penicillin (3.3%), and doxycycline (2.8%). Fish antibiotics with a high percentage of “likes” among human consumption-related reviews are as follows, ciprofloxacin (36.4%), amoxicillin (34.5%), and metronidazole (25.8%). Select reviews demonstrating potential human consumption are shown in Fig 2.

**Table 2:** Consumer reviews on fish antibiotics and proportion of reviews related to human consumption.

Fish antibiotic	Percentage of human consumption-related reviews, n_hr_/n_tr_^1^(%)	Percentage of Likes among human consumption-related reviews, n_hl_/n_tl_^2^ (%)	Percentage of Dislikes among human consumption -related reviews, n_hd_/n_td_^3^ (%)
Amoxicillin	28/1314 (2.1)	405/1173 (34.5)	7/8 (87.5)
Cephalexin	12/445 (2.7)	6/121 (5.0)	0/6
Ciprofloxacin	3/89 (3.4)	24/66 (36.4)	0/0
Clindamycin	2/40 (5.0)	3/16 (18.8)	0/0
Doxycycline	1/36 (2.8)	0/10	0/0
Erythromycin	1/12 (8.3)	0/0	1/1 (100)
Metronidazole	5/229 (2.2)	67/260 (25.8)	1/8 (12.5)
Penicillin	3/91 (3.3)	1/12 (8.3)	0/1
Sulfamethoxazole, Trimethoprim	0/32	0/16	0/0
Total	55/2288 (2.4)	506/1674 (30.2)	9/24 (37.5)

^1^ The percentage of human consumption-related reviews: the number of human consumption-related reviews (n_hr_)/the number of total reviews of the fish antibiotic (n_tr_).

^2^ The percentage of likes among human consumption-related reviews: the number of likes among human consumption-related reviews (n_hl_)/the total number of likes among all reviews (n_tl_).

^3^ The percentage of dislikes among human consumption-related reviews: the number of dislikes among human consumption-related reviews (n_hd_)/the total number of dislikes among all reviews (n_td_).

**Fig 3:**
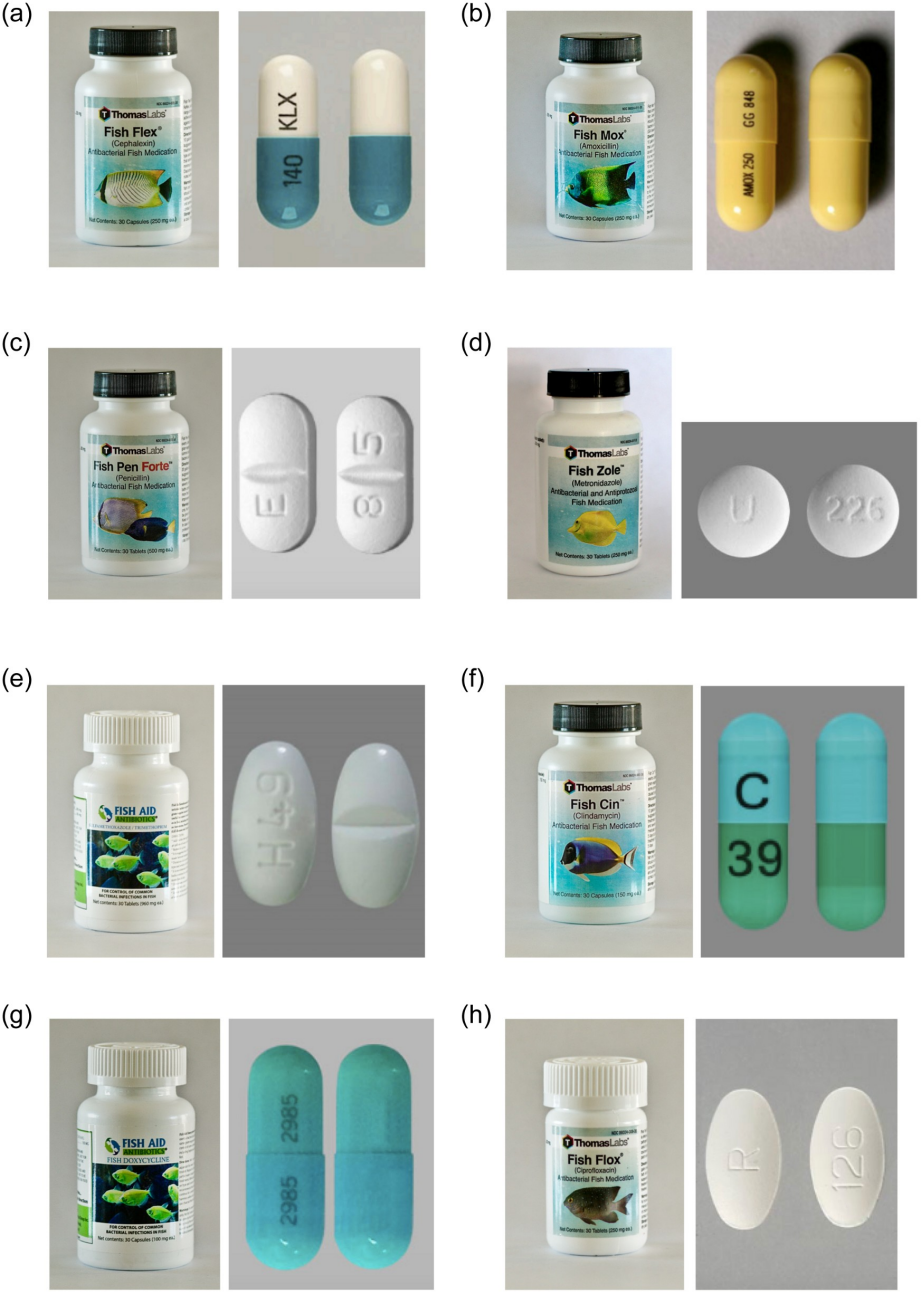
(3a-3h). Pill and capsule identification of 8 fish antibiotics. Fig 3a Generic Name: Cephalexin; Shape: oblong; Color List: green; white; Imprint Side 1: 140; Imprint Side 2: KLX; Strength Field Collection: 250mg; Dosage Form: Capsule; Route List: Oral; Bioequivalence Rating: AB; Rx/OTC: Rx (multiple source). Fig 3b Generic Name: Amoxicillin; Shape: oblong; Color List: yellow; Imprint Side 1: AMOX 250; Imprint Side 2: GG 848; Strength Field Collection: 250mg; Dosage Form: Capsule; Route List: Oral; Bioequivalence Rating: AB; Rx/OTC: Rx (multiple source). Fig 3c Generic Name: Penicillin V Potassium; Shape: oblong; Color List: white; Imprint Side 1: E; Imprint Side 2: 8 5; Strength Field Collection: 500mg; Dosage Form: Tablet; Route List: Oral; Bioequivalence Rating: AB; Rx/OTC: Rx (multiple source). Fig 3d Generic Name: Metronidazole (Systemic); Shape: round; Color List: white; Imprint Side 1: U; Imprint Side 2: 226; Strength Field Collection: 250mg; Dosage Form: Tablet; Route List: Oral; Bioequivalence Rating: AB; Rx/OTC: Rx (multiple source). Fig 3e Generic Name: Sulfamethoxazole and trimethoprim; Shape: oval; Color List: white; Imprint Side 1: H 49; Strength Field Collection: Sulfamethoxazole 800 mg and trimethoprim 160 mg; Dosage Form: Tablet; Route List: Oral; Bioequivalence Rating: AB; Rx/OTC: Rx (multiple source). Fig 3f Generic Name: Clindamycin (Systemic); Shape: oblong; Color List: light blue; light green; Imprint Side 1: C; Imprint Side 2: 39; Strength Field Collection: 150mg; Dosage Form: Capsule; Route List: Oral; Bioequivalence Rating: AB; Rx/OTC: Rx (multiple source). Fig 3g Generic Name: Doxycycline; Shape: oblong; Color List: light blue; Imprint Side 1: 2985; Imprint Side 2: 2985; Strength Field Collection: 100mg; Dosage Form: Capsule; Route List: Oral; Bioequivalence Rating: AB; Rx/OTC: Rx (multiple source). Fig 3h Generic Name: Ciprofloxacin; Shape: oval; Color List: white; Imprint Side 1: R; Imprint Side 2: 126; Strength Field Collection: 250mg; Dosage Form: Tablet; Route List: Oral; Bioequivalence Rating: AB; Rx/OTC: Rx (multiple source).

Among the 8 antibiotics purchased and compared to FDA-approved equivalents, all products were deemed consistent with human generic equivalents based on physical appearance. Chemical testing was not performed. Details regarding the physical products are available in Table 3 and Fig 3 (3a-3h). Erythromycin was not purchased because it was only available in a powder formulation.

**Table 3:** General information of 8 purchased fish antibiotics.

Generic name	Distributor	NDC	ID conformed	Labeler
Metronidazole	Thomas Labs^®^	86024-017-30	Yes	BLUE POINT LABORATORIES, UNICHEM PHARMACEUTICALS
Clindamycin	Thomas Labs^®^	86024-003-30	Yes	MYLAN INSTITUTIONAL
Amoxicillin	Thomas Labs^®^	86024-011-30	Yes	SANDOZ
Cephalexin	Thomas Labs^®^	86024-006-30	Yes	ORCHIDPHARMA
Penicillin	Thomas Labs^®^	86024-015-30	Yes	AUROBINDO PHARMA, GREENSTONE, NORTHSTAR RX, RISING PHARMACEUTICALS
Sulfamethoxazole/ Trimethoprim	Goldman Pharmaceutical Group Inc.	NA	Yes	MYLAN INSTITUTIONAL
Ciprofloxacin	Thomas Labs^®^	86924-008-30	Yes	PHYSICIAN PARTNER
Doxycycline	Goldman Pharmaceutical Group Inc.	NA	Yes	AVKARE

## Discussion

Reported self-medication with fish antibiotics documented in a viral tweet from 2017 drew significant market attention. The tweet directed readers to Amazon’s online fish antibiotics with reviewer comments suggesting human consumption [23]. Amazon has since discontinued the sale of fish antibiotics, although antiparasitic and antifungal fish medications remain available for purchase at the time of this publication. In the present study, there were numerous readily available websites selling fish antibiotics online. The findings of this study confirm that fish antibiotics can be freely purchased online without prescription in the US. Though, as anticipated, most customers are assumed to have bought the fish antibiotics for pets, 2.4% (55/2,288) of online customers potentially used them for self-medication based on the comments and reviews left on the products. These human consumption-related reviews contributed to 30.2% (506/1,674) of all “likes” received and 37.5% (9/24) of all “dislikes” received. Although the percentage of human consumption associated reviews is low, these reviews generate significantly more traffic compared to other reviews and comments. A number of studies have shown that purchasing behavior, intentions, and attitudes towards products can be influenced by the consumer ratings and reviews [24–26], and the most important factors affecting sales and attitudes are the valence and the volume of reviews [25–27]. Another study indicated that when overall “likes” are high, consumers are more likely to have higher brand involvement and purchase intention than when overall “likes” are low [28]. Customer reviews are essential, as it is estimated that approximately 58% of Americans conduct research about the products and services prior to buying them [29]. This suggests that human-consumption related reviews may encourage more people to use antibiotics marketed for fish for self-medication. Some social media platforms and websites also reported people were using fish antibiotics as a cheap alternative for self-medication and showed that this issue was reaching the lay public [21, 23, 30, 31].

In analyzing these human consumption related reviews, the authors felt that, consistent with other literature, multiple factors may contribute to patient self-medication with antibiotics, including affordability of physician visits, convenience of ordering and delivery to home, embarrassment associated with potential diagnosis, and lack of knowledge of antibiotic use and misuse. Surprisingly, one vendor customer service agent publicly expressed that fish antibiotics can be used in humans. There are certainly many potential hazards of self-medicating with fish antibiotics. As stated in a report issued by the FDA in 2018 [20], the safety and effectiveness of the antibiotics that are available in pet stores, or online had not been evaluated and have not been approved by the FDA. Recently, the FDA also released a statement to warn the public not to use chloroquine phosphate intended for fish [32] as treatment for COVID-19 in humans because these products sold for aquarium use have not been evaluated by the FDA [33]. All 8 fish antibiotics purchased had consistent physical findings with FDA-approved equivalents using pill identification, which is something that has drawn online attention in the lay public among advocates for self-treating using fish antibiotics [23, 30, 31]. However, regardless of identical physical appearance, these fish antibiotics may not meet FDA’s standards for purity and potency, and the handling and storage of these medications were unknown. The authors were unable to obtain further information regarding supply chain of the fish antibiotics from the vendors or suppliers.

Inappropriate use of antibiotics may cause a variety of adverse effects, including serious complications such as allergic reactions, organ dysfunction, or *Clostridioides difficile* infection [34–39]. Perhaps most concerning is that self-medication infers self-diagnosis, significantly increasing the risk for delayed, masked, or missed diagnoses [40]. This is largely concerning among communicable diseases, such as sexually transmitted infections (STIs), that may lend themselves to self-medication. In the present study, several product reviews of metronidazole specifically alluded to management of STIs including trichomoniasis and bacterial vaginosis. Inappropriate use of antibiotics without medical guidance may also increase the risk of the selection of resistant bacteria [41, 42]. While using fish antibiotics for self-medication may not contribute on a large scale to antimicrobial resistance, it certainly may impact the individual who chooses to self-medicate. Antibiotic consumption may can also impact future antibiotic choice if the patient ultimately seeks care for a particular infection [43, 44].

Some strategies may be needed to decrease self-medication and account for this use. Efforts on continuing public awareness of rational use of antibiotics appears warranted and necessary. The Centers for Disease Control and Prevention advocates for antibiotic awareness among patients and providers to improve antibiotic use and combat continued emergence of antibiotic resistance [45]. Upon knowledge of self-medication, clinicians should strive to determine patient specific motivators for self-medication and barriers to seeking care. These simple interventions may have long-term benefits for the patient to perhaps enhance medication access for the patient or manage other underlying reasons for self-medication. Continued discussion around the availability of antibiotics without medical professional advice is also warranted.

There are several limitations to this study. Firstly, this study is based on available website transactions and reviews, and thus it may not represent all vendors selling fish antibiotics. Additionally, the proportion of human consumption related reviews may be under- or overestimated due to consumer reluctance to disclose use of fish antibiotics for self-medication. Finally, our study focused only on English language websites and thus some websites in other languages were excluded.

This study is the first to objectively document the prevalence of fish antibiotics intended for human self-medication. Future studies should evaluate objective motivators behind self-medication with fish or other OTC antibiotics. Quality assurance and verification of stated contents of fish antibiotics is currently underway.

## Conclusions

Although infrequent, antibiotics intended for fish use are being purchased online without a prescription for self-medication to circumvent professional medical care. Reviews related to human consumption generate significant online traffic compared to reviews unrelated to human consumption. Patient education and clinician awareness of this phenomenon may help mitigate patient-specific utilization.

## Supporting information

S1 Table The list of websites included in the study. (DOCX)
