# Supplementary material for: Online availability of fish antibiotics and documented intent for self-medication
Source: PLoS One. 2020 Sep 3;15(9):e0238538. doi: 10.1371/journal.pone.0238538 (PMC7470343; doi:10.1371/journal.pone.0238538)
Supplement: S1 Table — (DOCX) [file pone.0238538.s001.docx]

**S1 Table. The list of websites included in the study**

| No. | Websites | URL | Date of browsing |
| --- | --- | --- | --- |
| 1 | Walmart PetRx | https://www.walmartpetrx.com | 8/29/2019 |
| 2 | chewy | https://www.chewy.com | 8/30/2019 |
| 3 | Allivet | https://www.allivet.com | 8/31/2019 |
| 4 | Revival Animal Health | https://www.revivalanimal.com | 8/31/2019 |
| 5 | HealthyPets | https://www.healthypets.com | 8/31/2019 |
| 6 | Valleyvet.com | https://www.valleyvet.com | 8/31/2019 |
| 7 | WAYOUT PETS | https://www.wayoutpets.com | 8/31/2019 |
| 8 | ThomasLabs | https://www.thomaslabs.com | 9/5/2019 |
| 9 | eBay | https://www.ebay.com | 9/9/2019 |
